# Experience of care from the perspectives of inpatients with palliative care needs: a cross-sectional study using a patient reported experience measure (PREM)

**DOI:** 10.1186/s12904-024-01494-4

**Published:** 2024-07-18

**Authors:** Gursharan K. Singh, Alison Mudge, Robyn Matthews, Patsy Yates, Jane L. Phillips, Claudia Virdun

**Affiliations:** 1 Centre for Healthcare Transformation, Faculty of Health, Queensland University of Technology (QUT), Brisbane, QLD Australia; 2 Cancer and Palliative Care Outcomes Centre, School of Nursing, Queensland University of Technology (QUT), Brisbane, QLD 4059 Australia; 3 Greater Brisbane Medical School, The University of Queensland, Herston, QLD Australia; 4 Department of Internal Medicine and Aged Care, Royal Brisbane and Women’s Hospital, Butterfield Street, Herston, QLD Australia; 5 Cancer Care Services, Royal Brisbane and Women’s Hospital, Butterfield Street, Herston, QLD Australia; 6 Research Centre for Palliative Care, Death, and Dying, College of Nursing and Health Sciences, Flinders University, Bedford Park, SA Australia; 7 Improving Palliative, Aged and Chronic Care Through Clinical Research and Translation (IMPACCT), Faculty of Health, University of Technology Sydney (UTS), Ultimo, NSW Australia

**Keywords:** Palliative care, Hospitals, Quality improvement, Quality of health care, Inpatients, PREMS

## Abstract

**Background:**

Improving palliative care for inpatients is urgently needed. Data from patient-reported experience measures (PREM) can assist in identifying areas for focused improvement. This study aimed to describe patient reported experience of care in inpatients with palliative care needs, to inform a baseline understanding of care experience and identify key areas for improvement.

**Methods:**

Cross-sectional study design where inpatients with palliative care needs were invited to complete ‘consideRATE,’ a patient reported experience measure of care, over six months in 2022. Inpatients with palliative care needs receiving care on an oncology, general medicine/renal and general medicine/respiratory ward (*n* = 3) at an Australian metropolitan hospital were screened for eligibility. Carers could provide proxy responses where inpatients were unable to participate. Descriptive statistics were used to analyse quantitative ratings, whilst free text responses were analysed using integrated thematic analysis.

**Results:**

One-hundred and twenty participants (108 patients and 12 carers) completed consideRATE. The questions with the highest number of ‘very good’ responses were attention to symptoms, attention to feelings and attention to what matters most; the questions with the lowest number of ‘very good’ responses was attention to patients’ affairs, what to expect, and the environment of care. Almost half (*n* = 57, 48%) indicated that attention to patients’ affairs ‘did not apply’ to their inpatient stay. Analysis of 532 free text responses across 8 questions highlighted the importance of feeling supported, feeling informed, feeling heard and navigating the clinical environment.

**Conclusion:**

Enabling inpatients with palliative care needs to provide feedback about their experience of care is one method of ensuring improvements matter to patients. Supporting clinical teams to understand and use these data to make tailored improvements is the next step in this multi-phase research.

## Key statements

### What is already known?

- High-quality care for inpatients with palliative care needs and their families is enabled when patients and families receive expert and person-centred care within an environment optimised to address their needs.
- Patient Reported Experience Measures (PREMs) can amplify the inpatients’ experience of care and is key to empowering innovation and improvement.

### What this paper adds

- This study amplifies the voice of acutely unwell inpatients with palliative care needs by providing patient reported experience of care data, using a brief, validated PREM to guide improvement for this population.
- Inpatients with palliative care needs rated attention to symptoms, attention to feelings and attention to what matters most as 'very good.' Almost half of respondents indicated that attention to patients' affairs 'does not apply' to their inpatient stay.
- Good quality palliative care is enabled when hospital clinicians ensure that inpatients feel supported, informed, and heard. This is influenced by the clinical environment where care is provided.

### Implications for practice, theory or policy

- Utilising a brief, validated patient reported experience measure, based on the priorities of the key stakeholder, provides meaningful data on the inpatient experience of receiving care. However, the PREM’s question relating to ‘attention to affairs’ may need modification in the inpatient setting.
- Patient reported experience data is gaining attention as a healthcare quality indicator, as it can provide insight into how patient-centred existing services are and identify areas for improvements in healthcare delivery.
- Understanding how to support clinical teams to use such data to make sustainable improvements in healthcare delivery is an important next step.

## Background

Approximately 30% of adult inpatients are living with an advanced life-limiting illness and are likely to benefit from a palliative approach to their care [1, 2]. Unfortunately, poor symptom management, ineffective communication and insufficient input into decision making are some of the reasons why optimal inpatient palliative care is not always provided [3–5]. Despite the global recognition for the last 30 years of the need to improve inpatient palliative care, how to drive focused and sustainable improvements remains elusive [6, 7].

Consistent evidence on what is important for high-quality care for inpatients with palliative care needs and their families exists [8–10]. Patients and families desire expert, person-centred care within an environment optimised to address their needs [11]. Measuring care experience for inpatients with advanced disease to identify improvement areas is noted to be one of 5 key drivers to enable sustained improvements in quality palliative care provision [12] (Table 1).

**Table 1:** Key drivers to support improved palliative care within the hospital context

*1. “Recognising and valuing palliative care as core business and a priority for inpatient care*
*2. Leadership at macro (policy), meso (hospital executive) and micro levels (ward) to develop systems and processes to enable optimal palliative care provision in accordance with consumer need*
*3. Measurement to inform quality assurance and identify targets for improvement*
*4. Innovation to co-design, with clinicians, administrators, other relevant experts and palliative care consumers, structures and processes that align with required patient and family-identified needs for optimal care; and*
*5.Targeted skill development to support clinicians and ancillary staff in their delivery of palliative care.” (p.205–206)* [12].

Patient Reported Experience Measures (PREMs) are one approach to measure experience to enable targeted improvements in quality inpatient palliative care [6, 13–15]. PREMs differ to patient satisfaction measures, which measure how well a patient’s expectations were met [16] and patient-reported outcome measures (PROMS) which measure the health status and wellbeing of patients at a single time point [17] and can be used to improve communication and shared decision making between patients and health professionals [18]. PREMs however, measure the patient’s experience of the healthcare service provided [19] and are defined as *“survey tools used to record patient perceptions about various elements of the healthcare they received’*(p.1) [20]. PREMS are increasingly attracting attention as a health care quality indicator and can provide insight into how patient-centred existing services are, as well as identifying areas for improvements in healthcare delivery [21]. Providing clinical teams with robust PREM data, tailored to areas of care that matter most to people with palliative care needs has the potential to empower innovation and improvement, but uptake into clinical practice has been slow [13, 19].

A recent qualitative study, led by members of this research team, asked inpatients with palliative care needs and their families about key requirements of a PREM to assess their experiences of hospital care [13]. Inpatients and their families noted the need for a PREM to be tailored to what matters most to the inpatient group, be brief, easy to use, and have space for free text responses [13]. Clinicians noted their need for data to be specific enough to inform practice or organisational changes [13]. Of all the PREMs designed for use with people with palliative care needs [6], consideRATE was the only one that meet these stated criteria. ConsideRATE is a reliable and valid PREM with eight brief and simple questions written in accessible English language [22] and has been found to be feasible and acceptable in the Australian hospital context (unpublished data, Virdun C, Button E, Phillips J, Saunders C, Yates P, Luckett T). No other similarly available measure of serious illness experience that is brief, simple or based solely on the priorities of people who are seriously ill currently exists [22]. This study aimed to describe patient reported experience data using consideRATE, from inpatients with serious illness likely to be in their last year of life, to inform a baseline understanding of care experience and identify key areas for improvement.

## Methods

### Design

A cross-sectional sub-study, using consideRATE [22], a validated PREM that has been found to be feasible and acceptable for use with palliative care inpatients [23]. This sub-study was undertaken as part of a larger, three-phase mixed methods study which sought to identify key enablers and barriers to optimal palliative care (Phase 1), design and test potential solutions (Phase 2), and understand feasibility and acceptability of using PREM data to drive change (Phase 3). Phase 1 included using a brief PREM tool to describe patient reported experience data from inpatients and carers (proxy measure), (which is reported here), as well as conducting in-depth staff interviews about the local context and current palliative care practices, which will be reported separately. These data from Phase 1 will be used to co-design and test tailored improvements in Phase 2.

### Setting and participants

#### Setting

The study was undertaken in three wards (oncology, mixed general medicine/renal and mixed general medicine/respiratory) in a publicly funded Australian tertiary metropolitan hospital. Each ward is served by several clinical teams, including consultant physicians with their teams of vocational and pre-vocational trainees and allied health teams including physiotherapists, occupational therapists, social workers, dietitians and speech pathologists. The hospital has mandated nursing ratios of one nurse to 4 inpatients on morning shifts. While the hospital does not have a designated palliative care unit, specialist consultative palliative care services are available to all inpatients. Wards are organized in a mixture of 4-bed, 2-bed and single rooms, and each ward has one small patient lounge area. Data collection occurred during a COVID-19 surge with visitor restrictions, face mask usage, and people with COVID-19 and their close contacts restricted from visiting.

#### Participants

Between June-November 2022, inpatients with serious, progressive illness, with a likely prognosis of < 12 months [1] were screened for eligibility. Adult inpatients (or their family members / carers ('carers')) admitted for > 24 h who had a complex, serious illness as defined by the Supportive and Palliative Care Indicators Tool (SPICT™) [24] with ≥ 2 general indicators of poor or deteriorating health and ≥ 1 clinical indicator of one or multiple life-limiting conditions were eligible to participate. Inpatients were ineligible if they had severe cognitive impairment, were clinically unstable, acutely distressed, or unable to communicate for other reasons. Translators were available to support those who did not speak English, however, translation use within this study was not required. Carers were welcome to assist inpatients as required (e.g., those with moderate cognitive impairment). As consideRATE has recently been validated for proxy scoring by carers (unpublished data, Nano JP, Stevens G, Elwyn G, Petrov L, Gowrishankar S, Robitaille S, Van Citters A, Nelson E, Wasp GT, MacMartin MA, Kirkland KB & Saunders CH.), if an inpatient was ineligible and a carer was present, the carer was invited to participate (proxy rating).

#### Recruitment

Approximately two days/week, the project facilitator screened all inpatients on participating wards for eligibility, prior to confirming with the nurse in charge. Eligible inpatients were provided with a study information sheet before consent was sought. Recruitment ceased after 40 participants from each ward completed consideRATE.

#### Instrument

Consenting participants were invited to report their care experience via a validated PREM, consideRATE [22], designed to measure perceived care experience for inpatients with serious illness. ConsideRATE’s eight questions are listed in Table 2. ConsideRATE strongly aligns with important areas for optimal quality care from inpatients’ perspectives [6, 10, 22]. A modified version of consideRATE [22] was used with a free-text option added per question.

**Table 2:** ConsideRATE’s eight questions [22]

How would you rate our attention to your physical problems? *things like pain, dry mouth or trouble breathing*
How would you rate our attention to your feelings? *things like feeling sad, worried or like a burden*
How would you rate our attention to your surroundings? *things like noise, light or warmth*
How would you rate our respect for what matters to you? *things like values, preferences about care or important activities*
How would you rate our communication about your plans? *things like medicines, procedures or place of care*
How would you rate our attention to your affairs? *things like financial planning and preparing enduring documents*
How would you rate our attention to what you can expect? *things like illness getting worse or time left to live*
Are there any other things you want to share?

#### Data collection

Inpatients (or carers) completed consideRATE in hard copy, rating each question (as very good, good, bad, very bad or does not apply) and providing optional free text responses for each item. Participants were invited to complete the survey independently or with assistance from the project facilitator, depending on their preference and need. Assistance included reading questions aloud and transcribing inpatients’ verbal ratings and feedback. All data were collected on hard copies prior to entry into Qualtrics™. Demographic data including age, gender, whether the participant had a non-English speaking background and diagnostic criteria as defined by the SPICT™ was collected for all participants.

### Data analysis

Participant demographics and question ratings were summarised using descriptive statistics. For each question, a response of ‘very good’ was scored as 4 points, ‘good’ was scored 3 points, ‘bad’ was scored 2 points and ‘very bad’ was scored 1 point, with answers as ‘does not apply’ excluded from the analysis. To determine the overall item scores and the overall score, a test for normality was performed and data which was not normally distributed were presented as median and interquartile range (IQR). Integrated thematic analysis [25] was applied to the free text responses, as these were led by specific questions within consideRATE. Free text responses which referred to care received outside of the inpatient stay were excluded from analysis. Qualitative analysis of free text responses was intended to provide a deeper understanding of the quantitative findings, and findings were analysed alongside each other [26]. A skilled qualitative researcher (GKS) and a senior research nurse (RM) with skills in research and administering consideRATE conducted the initial qualitative analysis. The researchers followed Braun and Clarke’s [27] thematic analysis method, by: a) familiarising themselves with the data independently (GKS and RM), b) double coding free text responses to generate initial codes independently and checking alignment after preliminary coding (GKS and RM), c) meeting with a third researcher (CV) to develop descriptive themes by categorising codes at the question level, d) reviewing congruence of the data across themes and questions (GKS, RM and CV) and e) defining and developing analytical themes and sub-themes (full research team). Credibility was achieved through two researchers checking coding alignment prior to coding all free text data, multiple researchers reviewing congruence of the data across the themes within consensus meetings, and the full research team defining and developing final themes. In practice, this coding was led by 2 researchers (GKS and RM) and consensus discussions were had within several research team meetings (GKS, RM, CV and AM). Participants’ exact words are used in this publication and the selection of quotes was based on the principle of authenticity [28]. Biases were discussed amongst the research team during reflexive discussions.

### Ethical considerations

Townsville Hospital and Health Service Human Research Ethics Committee (Reference number: HREC/2022/QTHS/84709) approved the study in May 2022. Prior to completion of consideRATE, study purpose and aim were discussed with participants, with participant anonymity highlighted. The need for participants to provide written, signed and dated informed consent documentation was waived by the Human Research Ethics Committee and instead, completion of consideRATE constituted consent.

## Results

Of the 841 inpatients screened, 504 met the inclusion criteria and were eligible for review using SPICT™, with 171 (34%) being eligible for study enrolment. Of those eligible, 120 (108 inpatients and 12 carers; 40 respondents from each ward) agreed to participate (participation rate 70%). Fifty-one eligible inpatients did not complete consideRATE because: they were not approached (*n* = 10), off ward for any reason (*n* = 34), discharged home (*n* = 3), asleep (*n* = 2), unwell (*n* = 1) or declined without reason (*n* = 1). Table 3 shows participant characteristics.

**Table 3:** Demographics of participants who completed the consideRATE tool (*n* = 120)

**Demographics – Inpatients**	***n*** **(%)**
Sex	
Male	62 (57%)
Female	46 (43%)
Age (Median (IQR))	70 (59–76)
Diagnostic group	
Malignant disease	49 (45%)
Non-malignant disease	57 (53%)
Malignant and non-malignant disease	2 (2%)
**Demographics—Carers**	
Sex	
Male	5 (42%)
Female	7 (58%)
Age (Median (IQR))	77 (68–84)
Diagnostic group of inpatients cared for by the carer	
Malignant disease	2 (17%)
Non-malignant disease	10 (83%)

### Ratings of care experience

Table 4 summarises data from the survey responses. Questions with the highest proportion of ‘very good’ responses were attention to symptoms (question 1), attention to feelings (question 2) and attention to what matters (question 4). Attention to patients’ affairs (question 6), attention to what you can expect (question 7) and attention to the physical environment (question 3) had lower proportions of ‘very good’ ratings (Table 4). Almost half (*n* = 57, 48%) of participants stated that the question about attention to patients’ affairs (question 6) ‘did not apply’ to their inpatient stay. All responses tended to be left-skewed, and median score was 3 (‘good’) for all questions.

**Table 4:** Patient (*n* = 108) and carer (*n* = 12) responses to the consideRATE survey (*n* = 120)

Question	**Rating **(***n***, %)					
	**Very good**	**Good**	**Bad**	**Very bad**	**Doesn’t apply**	**Median score (Interquartile range)**
**Q1 Symptoms**	57 (48)	57 (48)	5 (4)	0 (0)	1 (1)	3.0 (1.0)
**Q2 Feelings**	38 (32)	77 (64)	3 (2)	1 (1)	1 (1)	3.0 (1.0)
**Q3 Environment**	19 (16)	80 (67)	20 (16)	1 (1)	0 (0)	3.0 (0.0)
**Q4 What matters**	35 (29)	79 (66)	3 (2)	1 (1)	2 (2)	3.0 (1.0)
**Q5 Communication**	33 (28)	77 (64)	10 (8)	0 (0)	0 (0)	3.0 (1.0)
**Q6 Affairs**	12 (10)	49 (40)	2 (2)	0 (0)	57 (48)	3.0 (0.0)
**Q7 What to expect** ^a^	17 (14)	86 (72)	9 (8)	0 (0)	7 (6)	3.0 (0.0)

^**a**^ ***n*** = 119 respondents

#### Core elements ofquality care experience

**Fig. 1:**
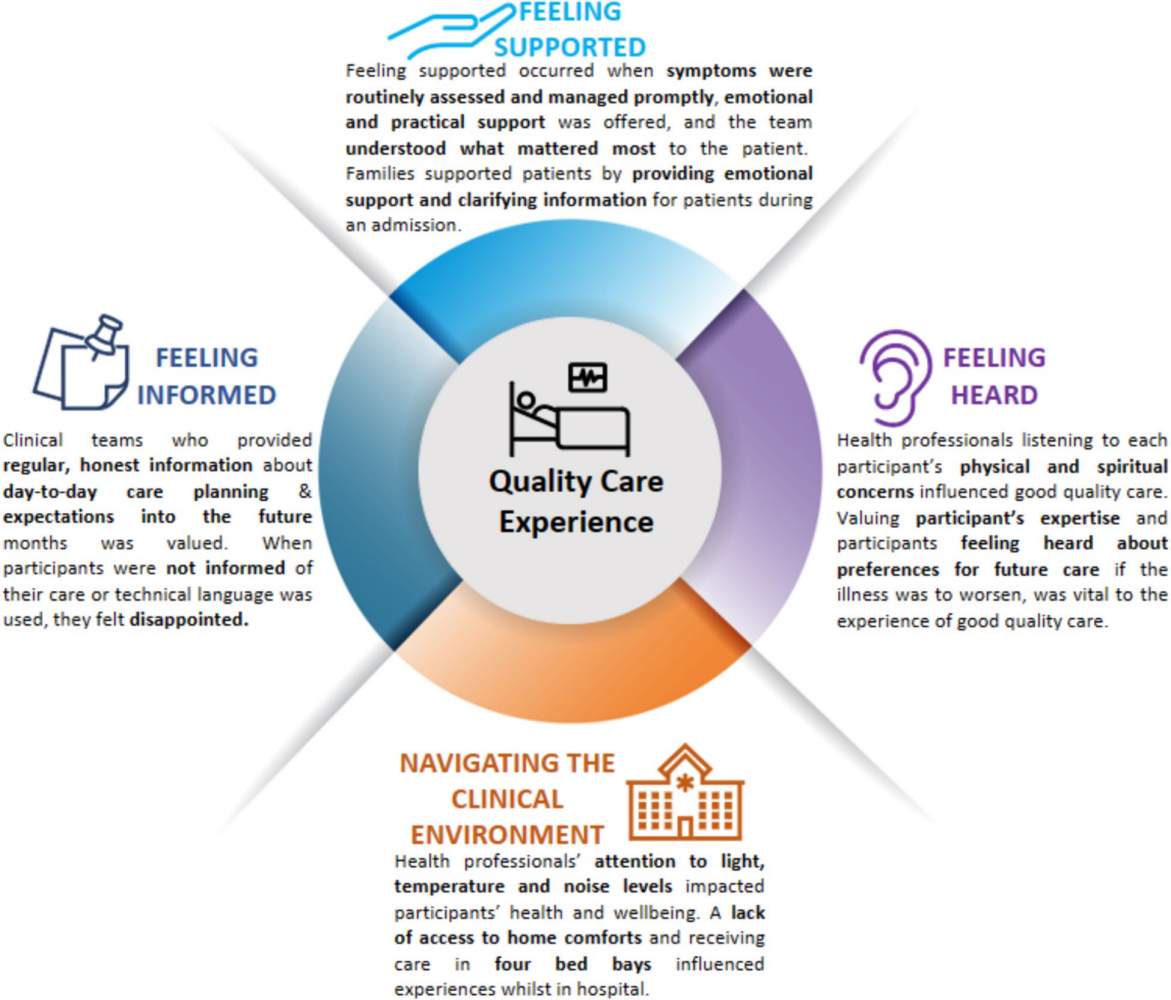
Key elements of quality inpatient palliative care experience

Of the 532 free text responses analysed across 8 questions, four themes influenced experiences of care quality including: 1) Feeling supported, 2) Feeling informed and 3) Feeling heard, referring to provision of care; and 4) Navigating the clinical environment, referring to the environment in which care is provided (Fig. 1).

#### Feeling supported

Participants felt supported when the hospital clinical team assessed and managed symptoms promptly, offered emotional and practical support, and understood what mattered most to patients. Families provided emotional support and clarified information for inpatients throughout an admission.

##### Regular assessment and prompt symptom management

The importance of timely symptom management, enabled by supportive care models and allied health input was highlighted. Participants described their distressing symptoms, and how they valued the clinical team regularly assessing symptoms and offering effective symptom management.

*“When I was getting short of breath, I had a team of people around me. They are providing me with oxygen at home so I can manage for a bit there with my family. I feel so much better than when I came in and I have this ward to thank for it.” (Patient, Male, 78, malignant illness)*

Allied health input and supportive care models that enabled good symptom management and improved quality of life were also valued.

*“Mum has been struggling to swallow well and has been coughing on anything she drinks, the speech pathologist has been working with us to improve this as it is really impacting her life.” (Carer of Female, 93, non-malignant illness)*

Participants spoke about the value of prompt symptom management. Some reported processes for charting medications, busy health professionals and understaffing resulted in delays in symptom management.

*“Nurses paged the doctor at night to get medications charted and a cannula put in to help my vomiting. It isn't their fault, but it took a very long time so I was unwell for quite a while. It feels like there is a lot of understaffing hospital wide.” (Patient, Female, 61, non-malignant illness)*

##### Receiving practical and emotional support from health professionals

Clinical teams’ provision of emotional and practical support reflected participants’ perception of how respectful clinicians were.

*“I feel it has been very respectful, I always receive support I am never left alone when I am upset or worried.” (Patient, Male, 68, malignant illness)*

Most appreciated the clinical teams’ emotional support after receiving distressing news or organising practical support at home:

*“I am seeing the social worker, psychologist and kidney supportive care - the nurses are very supportive too, I've had difficult news this week.” (Patient, Male, 49, non-malignant illness)*

*“The social worker is helping me find a nursing home, this is important because I need more help and support, I can't be at home. (Patient, Male, 70, non-malignant illness)​*

However, a few participants described situations where they felt unsupported as they were given difficult news alone.

*“You shouldn't tell people difficult news by themselves or without checking with them if they would like some support - always make sure they have the option of a support person so they aren't left alone …. it’s important to remember that these are people with lives and families.” (Patient, Male, 40, malignant illness)*

##### Supporting what matters to inpatients

Participants appreciated clinicians' paying attention to what mattered to them. This was enabled by checking in with participants:

*“There have been people who have checked in on me to make sure I'm not just lying here thinking about things. They offer magazines and talk to me.” (Patient, Female, 78, malignant illness)*

Participants also appreciated clinician’s being respectful of what matters to the inpatient and family and supporting future care planning.

*“The ACP [advance care planning] nurse came to see me - I have done my decision maker documents. It’s a good service to have someone checking in about this sort of paper work, you don't always know what to do with it.” (Patient, Female, 96, non-malignant illness)*

However some participants recognised that health professionals did not always prioritise what matters to them in the busy acute care environment.

*“They’ve got a job to do. It would be nice and it makes it easier when there is a focus on what matters to you but I understand there are other priorities to focus on like treatment plans, processes and procedures.” (Patient, Male, 74, non-malignant illness)*

##### The role of families in providing support

Participants confirmed the vital role of family members in providing emotional support, which could reduce the need for emotional support provided by clinicians.

*“I get a lot of emotional support from my family.” (Patient, Female, 46, malignant illness)*

Many family members provided support to the participant by being an intermediary to communicate care plans to participants and facilitate their understanding of the care.

*“They mostly talk to my daughter who helps me understand what is happening.” (Patient, Male, 81, non-malignant illness)*

#### Feeling informed

Most participants appreciated clinical teams who provided regular, honest information about day-to-day care planning and expectations of the future. This enabled participants to understand their treatment and make decisions about care. When health professionals did not inform participants of their care or used technical language, participants felt disappointed and felt clinical teams were disjointed and siloed.

##### Providing honest and regular information

Participants valued being regularly informed with up-to-date information by their clinical team and having opportunities to ask questions and clarify information.

*“I can't fault the care and I feel like I have been informed by all the people who come to see me, I am kept up to date, the communication has been open and I can clarify what I need to.” (Patient, Male, 71, malignant illness)*

*“They always clarify if we understand what they're saying. They [my family] are always involved by staff and feel they can ask questions. It has made it easier for them to accept/understand what's going on with me.” (Patient, Female, 41, malignant illness)*

Participants appreciated honesty when they asked about future expectations of their illness so they could be prepared for the future.

*“I appreciate honesty so I ask the tough questions. It's not always easy to hear the answers but I want to be prepared.” (Patient, Male, 66, non-malignant illness)*

When health professionals took the time to speak openly and explain the overall care plan, it enabled participants to make informed decisions about their care.

*“Lots of people have been involved in Mum's care - I have found talking to palliative care helpful to bring it all together and understand the overall plan.” (Carer of Female, 75, malignant illness)*

Participants noted the difficulties they experienced when clinical teams do not provide clear communication, contradict each other, or show a lack of integrated care planning and provision.

*“It fluctuates and can be disjointed. Doctors can contradict each other and vary depending on who's in charge. I don't really understand the global plans because there are so many teams involved - I feel disappointed that there is a lot of "lets watch and see how it goes". (Patient, Male, 63, malignant illness)*

#### Feeling heard

Good quality care experience was influenced by health professionals listening to participants’ physical and spiritual concerns. Valuing and acknowledging participants’ illness and symptom expertise was important for shared decision making. Feeling heard about preferences for future care was vital to the experience of good quality care.

##### Listening to physical and spiritual concerns

Listening to and respecting participants’ spiritual beliefs when making decisions about care was valued and influenced the perception of good quality care.

*“I believe in God - the doctor I was talking to about my faith, he listened to me. It may not have been his belief but he sat and listened. The people that work here are a gift - the care has been magnificent.” (Patient, Male, 78, malignant illness)*

Participants spoke about their symptom and illness expertise and the importance of being involved in the decision making around diagnostics and the care process. Listening to participants when they expressed symptom and illness concerns enabled participants to feel cared for and confident in the clinical team.

*“I am a complex, long-term patient because of my kidney failure. I have been in hospital a lot. It can be frustrating when you feel you're not being listened to about what you think is wrong with you and it can take a lot of time to get to the bottom of it. I feel like I wasn't believed and, in the end, what I suspected was wrong with my back has shown to be correct on the imaging. I understand I am not a professional and appreciate there are processes and procedures, but I do know my body and as I said, I have been in and out of hospital over the years a lot with issues with my health. Sometimes it feels like my voice isn't heard.” (Patient, Female, 44, non-malignant illness)*

For this reason, participants valued the opportunity to complete the PREM tool:

*“I've really appreciated being asked about my experience - I have had a good experience but it is nice to be spoken to about it like this and be able to give feedback to improve care.” (Patient, Female, 61, non-malignant illness)*

##### Feeling heard about care preferences if their illness worsens

Participants expressed the importance of being and feeling heard about preferences for care if their illness worsened. The focus on quality of life and not being burdensome to family was viewed to be important.

*“I've spoken to my doctors about what I want if my illness gets worse like what is happening now. I don't want to suffer. We had a family meeting and the discussion was open and focused on what I wanted from here. Right now I feel like my quality of life is poor. I'd like to focus on comfort and I expressed that. I know they are going to withdraw the dialysis and I understand that I won’t have a long time left from there but I will be kept comfortable.” (Patient, Male, 76, non-malignant illness)*

Many spoke about completing not for resuscitation documents with their doctors and used this as an opportunity to discuss preferences for a quality-of-life focus if their illness worsened.

*“My quality of life is most important to me. I know my family want me to prolong my life and have as much time as possible but I don't want to live if my life isn't quality. I don't want to be a burden on my family. I have completed a not for resuscitation document with my doctors so that has been discussed a bit then.” (Patient, Male, 75, non-malignant illness)*

#### Navigating the clinical environment

This theme reflected the realities of receiving care within an acute environment and its impact on care quality. Health professionals’ attention to light, temperature and noise levels impacted participants’ health and wellbeing. A lack of access to television and kitchen appliances removed comfort and connection. Being in bays with other inpatients also influenced participants’ inpatient experiences.

##### Attention to light, temperature and noise level is appreciated

Participants understood the busy nature of the hospital when tending to unwell inpatients resulted in noise.

*“It is loud and busy in here and there are lots of confused people but I understand this is a hospital and people are quite unwell.” (Patient, Male, 86, malignant illness)*

However, participants appreciated when healthcare providers were attentive to light, temperature and noise, as this was important for their comfort and wellbeing.

*“The air conditioning feels very dehydrating - it is all the perfect storm for a migraine which I have suffered from for a long time. I feel people should be asked about this on admission.” (Patient, Female, 61, non-malignant illness)*

##### Lack of access to home comforts

Participants spoke about the lack of access to television, noting televisions provided connection to the world, a way to pass time while in hospital, and enabled a distraction from living with serious illness.

*“You can't watch the news to see what is going on because there are no TVs - I've already missed so much this year with being unwell. It's not fair to have nothing to watch to give you some kind of pleasure…People sit in their room all day listening to their own thoughts.” (Patient, Female, 73, non-malignant illness)*

A lack of access to simple kitchen appliances removed a sense of comfort, limited independence and detracted from a participant’s sense of self.

*“It would be really good to have access to a kettle to make a cup of tea, sometimes you don't want to bother the staff …. but it can be something small like that that is comforting and makes you feel human, just enjoying a cup of tea.” (Patient, Female, 44, non-malignant illness)*

##### Receiving care within four bedded bays

Participants noted their appreciation for ancillary staff and attention to cleanliness and hygiene when sharing common areas with other inpatients, although their expectations were not always met.

*“The toilet hasn't been cleaned properly from a man in the bay preparing for a colonoscopy.” (Patient, Female, 78, malignant illness)*

Preferences for bays with multiple inpatients varied. Some participants valued being with others for social connection.

*“I'm a social person, not private - I like having the curtains pulled back and chatting.” (Patient, Female, 71, malignant illness)*

However, some struggled with the lack of privacy due to their usual living arrangements.

*“The lack of privacy is very hard when you're unwell in these 4 bed rooms. I'm not used to it, I live alone.” (Patient, Female, 75, non-malignant illness)*

## Discussion

This study describes PREM findings from 120 inpatients assessed as likely to be in the final 12 months of life. Survey responses indicated positive ratings for attention to symptoms, attention to feelings, and attention to what matters most to patients. Free-text responses similarly reflected the importance of regular assessment and prompt symptom management, receiving practical and emotional support from multiple health professionals working as a team, and supporting what matters to inpatients. These findings are similar to previous studies in the Australian hospital setting, where spiritual and emotional support, communication between the participant and health professional about their care options, and effective symptom management were important [11, 29]. Similarly, analysis of free text responses from a Canadian study of patient care experience for people in the community with cancer noted the importance of attention to symptom, emotional and spiritual concerns and being listened to by health professionals [30].

Less positive responses were seen for the questions relating to what to expect. In the free text responses, while most participants felt informed and listened to about their current concerns, and generally welcomed honest dialogue about the future, prognostic communication was often vague, focussed primarily on resuscitation orders, were contradictory, or showed a lack of integrated care planning and provision. The quality of prognostic communication within clinical settings can be due to institutional and healthcare system barriers such as clinical staff shortage and high workload [31]. Clear, consistent information provided by attentive health professionals can improve shared understanding of prognosis within and between treating teams and increase inpatients’ confidence in their care [29]. Numerous studies on patient and healthcare professional communication emphasise the importance of “whole person” knowledge, including prognostic information, in increasing trust in the clinical processes and reducing complications during inpatient stays [32, 33].

In the free text responses, study participants outlined recommended improvements to the clinical environment such as temperature, noise and access to home comforts, and reported both positive and negative experiences of receiving care in bays with multiple inpatients. A hospital’s built environment can impact on a inpatient’s experience and outcomes [34]. Positive design features for optimal palliative care delivery include privacy and homeliness, access to private spaces, outdoor green spaces and lounge areas [35]. In particular, private spaces for receiving bad news and that are conducive for reflection are important to palliative patients and families [36, 37]. However, hospital facilities often lack these features when constructed and are difficult to modify [34]. Nonetheless, small actions that enable inpatients to influence their environment such as encouraging familiar items from home, providing access to hot drinks, and controlling sound, lighting and temperature can help to maintain a sense of control and normality [38], which can improve outcomes for inpatients [39].

This study is unique as, to the best of our knowledge, this is the only reported study describing patient reported experience data for inpatients likely to be in their last year of life with an acute care admission. The selection of the PREM, consideRATE, was driven by key stakeholders, including patients and health professionals, in accordance with their priorities for optimising care [22, 40]. Providing a method to amplify the care experience of inpatients with palliative care needs is both unique and important in informing improvements in care within complex acute hospital environments. The good response rate (70%) obtained in this study highlights that it is possible to collect meaningful patient reported experience data from inpatients with palliative care needs, despite their high levels of illness, disability and cognitive load, using a brief, simple PREM. Our data also highlights some challenges with the PREM tool scoring in this inpatient setting. Almost half of participants scored the question about attention to affairs as ‘does not apply’, which was excluded from the item median score analysis. This finding may reflect different expectations in acute care inpatients and suggests that the tool may need to be tailored for different settings. Data for the remaining items were left skewed so we reported median and interquartile range; the predominance of ‘good’ scores across items meant that median item scores were 3 across all domains. Discriminant validity of consideRATE has previously been tested using simulated stories which may have provided more normally distributed scores and reported mean scores [23]; further testing in real-world patient populations is warranted. To inform improvement in Phase 2 of the larger study, we have decided to present score categories (as shown in Table 4) rather than medians to our local clinicians, accompanied by exemplar positive and negative quotes, to stimulate reflection and solution generation. The findings of the larger three-phase study will provide insights into using PREMs to drive improvements.

### Strengths and limitations

A strength of this study was the high response rate, likely facilitated by inpatients being approached by a research nurse in relation to PREM completion with assistance offered and the use of a tailored, short and easy to understand instrument. The PREM data within this study provide information on inpatients’ care experiences which will be used to drive service improvements [41]. While consideRATE does not focus on every aspect of the inpatient palliative care experience, it is the only PREM which is based solely on the priorities of people who are seriously ill and meets the key requirements of a PREM for use in this inpatient population. However, as consideRATE does not contain a ‘neutral’ option, purposely designed to encourage participants to select a rating, it can lead to a positive skew in ratings, as evidenced by a large percentage of participants selecting ‘good.’ A small number of carers provided proxy ratings of the inpatient experience as opposed to their actual carer experience.

## Conclusion

Understanding inpatient care experience is a critical first step to improved palliative care for people hospitalised in their last year of life. This study demonstrated that good quality inpatient experience is enabled when inpatients feel supported, informed, and heard. This is influenced by the clinical environment where care is provided. Improving communication about prognosis with inpatients and within the multidisciplinary team, and improving the clinical environment have great potential to optimise the experience of care at this important phase of a person’s life. The next phases of this research will utilise the PREM data to co-design and test locally tailored improvements and evaluate the feasibility and acceptability of this approach to drive improvements, and the impact on patient-reported experience.

## Data Availability

In accordance with the study’s ethical regulations and in the interest of patient and carer confidentiality and anonymity, data and analysis material related to this study will not be made publicly available. Requests for deidentified data (quantitative and qualitative) can be directed to the lead investigator for this study: claudia.virdun@flinders.edu.au
